# Enhancing medication error reporting through interprofessional education: analysis of Medwatch reporting accuracy and completion rates between teams and individuals

**DOI:** 10.1186/s12909-025-07349-7

**Published:** 2025-05-22

**Authors:** Aline H. Saad, Rehab Bondok, Farah Sayeg, Brian J. Barnes, Caitlin E. Rukat, Candice Garwood, Diane L. Levine

**Affiliations:** 1 Director of Faculty Development and Coordinator of Interprofessional Education, Detroit, Michigan, USA; 2 Pharmacy Practice Department, Eugene Applebaum College of Pharmacy and Health Sciences, Wayne State University, 259 Mack Ave, Suite 2190, Detroit, MI 48201 USA; 3 Professor (Clinician-Educator), Director, Curricular Integration-UME; UME Clerkship Director, Vice Chair for Medical Education. Department of General Medicine, Wayne State University, School of Medicine, 540 E. Canfield Ave., Detroit, MI 48201 USA

**Keywords:** Interprofessional education, Medication safety, MedWatch reporting, Medical students, Pharmacy students, Interprofessional teams, Patient safety, Healthcare education

## Abstract

**Background:**

Each year, the Food and Drug Administration receives over 2 million adverse event and medication error reports, which are likely underreported. Interprofessional education (IPE) is well positioned to provide team-based training regarding medication safety and related reporting tools. This study evaluated the effectiveness of a single IPE session designed to improve the completion and accuracy of healthcare professional students’ reporting of medication errors.

**Methods:**

An IPE session, with medical and pharmacy students, presented a case report involving a medication dispensing error that resulted in a patient’s death. The session included three components: the case presentation; a discussion of the implications of the medication error on the patient, family, and care providers; and a hands-on activity where students practiced error reporting using a simulated MedWatch platform. The students’ reports were analyzed for completeness and accuracy, based on data available from the case presentation. Individual versus team submissions across disciplines were compared.

**Results:**

Of the 701 participants who completed the session between 2021 and 2024, 225 submitted the simulated MedWatch report (32% response rate). This final sample included 111 medical students, 53 pharmacy students, and 61 interprofessional teams. The median form completion rate for teams was 88.9% compared to 55.6% for individuals. Teams demonstrated higher form accuracy rates (66.7%) compared with individuals (38.9%). Students agreed that practicing the reporting of an adverse drug event was a useful activity, while pharmacy students (*p* = 0.014) and teams (*p* = 0.043) felt more confident reporting an adverse drug event than medical students after this activity.

**Conclusion:**

Following an IPE training session focused on error reporting, we observed that team-based submission of MedWatch forms resulted in improved completion and accuracy rates. Integrating an interprofessional training session focused on medication safety and error reporting in health professionals’ curricula appeared to be effective in the short term. Longer term studies are necessary to determine the impact and durability of this training.

**Clinical trial number:**

Not applicable.

**Supplementary Information:**

The online version contains supplementary material available at 10.1186/s12909-025-07349-7.

## Background

Since the publication of “To Err is Human” in the year 2000, significant efforts have focused on improving the safety of our healthcare systems and reducing medication errors [1]. In 2016, Makary and Daniel reported that medical errors, including medication errors, could be leading to more than 250,000 deaths annually [2]. 10% of hospitalized patients will be impacted by a medication error [3]. In outpatient settings, approximately 530,000 injuries occur yearly due to medication errors [4]. A medication error as defined by the National Coordinating Council for Medication Error Reporting and Prevention is “any preventable event that may cause or lead to inappropriate medication use or patient harm while the medication is in the control of the health care professional, patient, or consumer” [5]. Overall, 41% of US citizens report experiencing or knowing someone who experienced a medication error [6]. In fact, the Food and Drug Administration (FDA) receives over 2 million adverse event and medication error reports every year [7].

The number of medication errors occurring in various health care settings is likely underreported [8–10]. Reporting medication errors provides an opportunity for improving patient and health care systems safety and care quality by learning lessons from the errors and devising interventions to deliver safer medication management systems. However, many barriers to reporting have been identified. These include fear of reprimand, reduced patient trust, lack of appreciation of the importance of reporting, and lack of support from supervisors [11, 12]. Additionally, the process of error reporting is not standardized across health systems and is time-consuming. Many of the health care providers remain unfamiliar with their institutions’ reporting platforms and policies related to the process [13, 14]. In fact, health systems employ different reporting platforms; some are internally developed while others are adopted from nationally available tools such as the MedWatch reporting by the FDA or the Medication Error Reporting Program (MERP) by the Institute for Safe Medication Practice (ISMP) [15, 16].

Finally, health professional curricula seldom provide students with training related to medication error reporting prior to practice. While patient safety competencies have been developed, teaching medication error reporting remains inconsistent across disciplines, years, and tools prior to practice [17–19]. Interprofessional education (IPE) offers a platform for medical and pharmacy students to collaborate, enhancing their understanding of safe medication prescribing, dispensing, administration, and error prevention [20, 21]. Recent literature demonstrates the effectiveness of interprofessional activities in increasing students’ knowledge in patient safety competencies [22, 23]. In fact, the role of teamwork and communication in preventing errors drive the design of many of these activities. However, little is known about how interprofessional team education would impact medication error reporting. Accordingly, the primary objective of this study was to assess the effectiveness of a single IPE session focused on improving the completion and accuracy of healthcare professional students’ reporting of medication errors. A secondary objective assessed participants’ value of the session and its impact on their confidence in reporting medication errors.

## Methods

### Setting

An IPE session, involving medical and pharmacy students, presented a case report involving a patient death caused by a medication dispensing error. The patient received the wrong tablet strength of warfarin, prescribed for anticoagulation in the setting of atrial fibrillation. The patient experienced a hemorrhagic stroke resulting in their death. The session included three components: (1) a case presentation detailing an error in dispensing an anticoagulation medication that led to the death of the patient, (2) discussion of the role of error disclosure and the implications of medication errors on the patient, family, and care providers, and (3) a hands-on optional activity where students reported the error using a simulated MedWatch form [20]. The simulated reporting form replicated the questions included on the MedWatch FDA reporting form and was administered as a Qualtrics survey hosted on the University’s server. Ten minutes were allotted for this activity. Students were provided additional time to complete the simulated MedWatch survey, if needed.

### Study population

The study population consisted of third year medical and pharmacy students attending a large urban public university from 2021 to 2024. The session was integrated in medical and pharmacy courses, so attendance was mandatory for all students. Between 2021 and September 2023, the session was offered virtually to students and the medication error reporting was completed individually by pharmacy and medical students. For the remainder of 2023 and 2024, the session was transitioned to in-person delivery and the error reporting was completed in interprofessional teams, each comprised of three medical students and one pharmacy student. Students or teams that designated their fields of study and completed at least one survey question were included in the study. All data collected in Qualtrics were de-identified. This study was reviewed by our Institutional Review Board (IRB) and qualified for exemption (IRB# 2024 118).

### Statistical analysis

The primary objective of this study was to identify if medical students, pharmacy students, and teams differed in their completion and accuracy of the medication error reporting activity. Eighteen questions in the MedWatch form (deemed relevant to the case report by the investigators) were assessed for completion and accuracy (see Table 3 for a list of the questions). Completion was defined as any response, regardless of whether it was correct. Accuracy was defined as correct responses only. Completion and accuracy were calculated for each of the 18 questions individually, as well as for the survey as a whole. Overall completeness was calculated by (number of complete responses/18) * 100. Overall accuracy was calculated by (number of correct responses/18) * 100.

Descriptive statistics of completion and accuracy were reported for each of the 18 questions among medical students, pharmacy students, individuals regardless of field of study (medical + pharmacy students combined), and teams using N (%). To reduce the likelihood of a type 1 statistical error, we selected four of 18 questions on the report for conducting inferential statistics (two-sided alpha was set a priori at 0.05). Accuracy of each of the four questions was compared among (1) medical students vs. pharmacy students vs. teams and (2) individuals regardless of field of study (i.e. medical + pharmacy students combined) vs. teams using the chi-square test of independence.

The distributions of overall accuracy and completeness were assessed and found to be skewed. Therefore, we compared median (+/- interquartile range) overall completeness and accuracy rates among the following groups: (1) medical students vs. pharmacy students vs. teams using the Kruskal-Wallis test and (2) individuals regardless of field of study (i.e. medical + pharmacy students combined) vs. teams using the Mann-Whitney U test. When a significant difference was identified between medical students, pharmacy students, and teams with the Kruskal-Wallis test, we conducted Dunn’s test with a Bonferroni correction (raw p-value divided by the number of comparisons being made) as a post-hoc analysis to determine which pairwise comparisons differed [24].

Since the interprofessional education session took place during the COVID-19 pandemic, instruction was delivered virtually using a videoconference platform (Zoom Communications, San Jose, CA) in 2021 and 2022, and returned to in-person in 2023 and 2024. The timing of return to in-person instruction coincides with the introduction of teams to the education session. To ensure any differences found between individuals and teams were not confounded by method of instruction (virtual vs. in-person), we compared median (+/- interquartile range) overall completeness and accuracy among individuals who completed the assessment virtually in 2022 to individuals who completed the assessment in-person in 2023, thus excluding all teams from the comparison and allowing us to evaluate the impact of instruction delivery method (i.e. virtual vs. in-person) on completeness and accuracy.

A secondary objective of this study was to evaluate students’ perceptions of the educational activity. Two questions were added to the MedWatch form that asked if the student (1) found this activity useful and whether they (2) felt more confident in reporting an adverse drug event after completing this activity. Responses were on a five-point Likert scale with response options ranging from strongly agree to strongly disagree. Median (+/- interquartile range) responses among medical students, pharmacy students, and teams were compared using the Kruskal-Wallis test and followed by Dunn’s test with a Bonferroni correction if there were significant findings [24].

All analyses were conducted using SAS version 9.4 and RStudio version 2023.12.1.402. All figures were generated using SAS version 9.4 and Microsoft Excel version 2406.

## Results

There were 701 students that participated in the IPE session across the four years of our study period. Of these 701 students, 430 did not provide information on their field of study and were therefore excluded from this analysis. There were 6 teams that provided information on field of study but did not answer any questions in the survey, resulting in their exclusion from the study. The final sample size was 225, consisting of 111 medical students, 53 pharmacy students, and 61 teams (32.1% response rate). Among the 61 teams participating in the simulated MedWatch reporting, 46 (75%) delegated a medical student to be their scribe.

### Assessment of overall completeness and accuracy

A significant difference in median assessment **completeness** rates between medical students (55.6%), pharmacy students (55.6%), and teams (88.9%) was found (*p* < 0.0001, Table 1). Teams had higher median completeness rates than medical students (*p* < 0.0001) and pharmacy students (*p* < 0.0001, Table 1). When comparing teams to individuals regardless of field of study, the association remained with teams having a median completeness of 88.9% compared with 55.6% for individuals (*p* < 0.0001, Table 1).

**Table 1:** Median completion and accuracy rates in the study population

				Group			
		Medical Students *N* = 111	Pharmacy Students *N* = 53	Individuals (Medical + Pharmacy Students) *N* = 164	Teams *N* = 61	*P*-value	
**Overall Completeness %** Median (Q1– Q3)		55.6% (44.4–61.1)	55.6% (50.0–61.1)	55.6% (47.2–61.1)	88.9% (77.8–94.4)	Medical Students– Teams: <0.0001^*^ Pharmacy Students– Teams: <0.0001^*^	
						Individuals– Teams: *<0.0001*^*†*^	
**Overall** **Accuracy %** Median (Q1– Q3)		33.3% (27.8–44.4)	38.9% (33.3–44.4)	38.9% (27.8–44.4)	66.7% (55.6–72.2)	Medical Students– Teams: <0.0001^*^ Pharmacy Students– Teams: <0.0001^*^	
						Individuals vs. Teams: *<0.0001*^*†*^	

* P-value reported for Dunn’s test following the Kruskal-Wallis test (comparing medical students vs. pharmacy students vs. teams)

^†^ P-value reported for the Mann-Whitney U test (comparing individuals vs. teams)

Repeating the previous method for assessing **accuracy**, we found similar results. A significant difference in median assessment accuracy rates between medical students (33.3%), pharmacy students (38.9%), and teams (66.7%) was found (*p* < 0.0001). Teams had higher median accuracy rates than medical students (*p* < 0.0001) and pharmacy students (*p* < 0.00001, Table 1). When comparing teams with individuals, regardless of field of study, the association remained with teams having a median accuracy rate of 66.7% compared with 38.9% for individuals (*p* < 0.0001, Table 1).

To ensure the greater rates of completeness and accuracy observed among teams was not due to a change in delivery instruction method, we compared completeness and accuracy among individuals that took the assessment virtually in 2022 with individuals that took the assessment in-person in 2023. The median completeness rate between virtual and in-person individuals was identical (both 55.6%, *p* = 0.64, Table 2). There was no significant difference in median accuracy, with virtual individuals having a median accuracy rate of 38.9% and in-person individuals having a median accuracy rate of 33.3% (*p* = 0.15, Table 2).

**Table 2:** Completeness and accuracy rates by instruction delivery method among those that took the assessment individually

		Virtual Instruction Individuals 2022 *N* = 113	In-Person Instruction Individuals 2023 *N* = 28	*P*-value*
**Completeness %** Median (Q1– Q3)		55.6% (44.4–61.1)	55.6% (44.4–61.1)	0.64
**Accuracy %** Median (Q1– Q3)		38.9% (27.8–44.4)	33.3% (22.2–41.7)	0.15

* P-value reported for the Mann-Whitney U Test

### Assessment of individual simulated Medwatch report questions

Descriptive statistics of completeness and accuracy for each of the eighteen questions (Q) can be found in Tables 3 and 4, respectively. Teams had the highest completion and accuracy rates for 14 out of the 18 questions. When students were asked to list laboratory tests relevant to the adverse event (Q13_2), 67% of teams answered accurately, compared with 13% of pharmacy students, 10% of medical students (*p* < 0.0001, Table 4), and 11% of individuals regardless of field of study (*p* < 0,0001, Table 4). When students were asked to identify the indication for using the drug (Q21), 69% of teams answered accurately, compared with 53% of pharmacy students, 39% of medical students (*p* = 0.0007, Table 4), and 43% of individuals regardless of field of study (*p* = 0.0007, Table 4). When students were asked to identify the type of report required (Q8), 2% of teams answered accurately, compared with 9% of pharmacy students and 14% of medical students (*p* = 0.026, Table 4), and 13% of individuals regardless of field of study (*p* = 0.012, Table 4). No significant differences were observed between teams, medical students, and pharmacy students, or teams and individuals in accuracy of responses when asked to identify the outcome attributed to the adverse event (Q9).

**Table 3:** Completion rates for each of the 18 questions assessed

		Question Completion *N* (%)			
		Medical Students *N* = 111	Pharmacy Students *N* = 53	Individuals (Medical + Pharmacy Students) *N* = 164	Teams *N* = 61
Question					
Q2: Patient identifier		85 (77)	40 (75)	125 (76)	50 (82)
Q8: Type of report		109 (98)	53 (100)	162 (99)	60 (98)
Q9: Outcome attributed to adverse event		109 (98)	53 (100)	162 (99)	58 (95)
Q11: Was this event associated with product use or treatment under Emergency Use Authorization (EUA)?		105 (95)	51 (96)	156 (95)	58 (95)
Q12: Describe the event, problem, or product use/medication error		106 (95)	53 (100)	159 (97)	58 (95)
Q13_2: Relevant laboratory test(s)		18 (16)	8 (15)	26 (16)	45 (74)
Q13_3: Results of the above test(s)		18 (16)	5 (9)	23 (14)	44 (72)
Q13_7: Please provide the descriptive results of the test or procedure. Include results, analyses, and evaluations.		8 (7)	5 (9)	13 (8)	14 (23)
Q14: Other relevant history, including preexisting medical conditions (e.g., allergies, pregnancy, smoking and alcohol use, liver/kidney problems, etc.)		69 (62)	35 (66)	104 (63)	58 (95)
Q19_1: Name of suspect product		103 (93)	48 (91)	151 (92)	60 (98)
Q19_2: Strength of suspect product		4 (4)	3 (6)	7 (4)	54 (89)
Q19_3: Units for the strength of the suspect product		0 (0)	0 (0)	0 (0)	50 (82)
Q19_9: Frequency of administration of the suspect product		1 (1)	0 (0)	1 (1)	43 (70)
Q19_10: Route of administration of the suspect product		0 (0)	0 (0)	0 (0)	42 (69)
Q21: Diagnosis for use (indication)		76 (68)	35 (66)	111 (68)	51 (84)
Q22: Product type (OTC, compounded, generic, biosimilar)		68 (61)	46 (87)	114 (70)	56 (92)
Q24: Event abated after use stopped or dose reduced?		85 (77)	38 (72)	123 (75)	52 (85)
Q25:Event reappeared after reintroduction?		86 (77)	39 (74)	125 (76)	52 (85)

**Table 4:** Accuracy rates for each of the 18 questions assessed

	Question Accuracy *N* (%)				
		Medical Students *N* = 111	Pharmacy Students *N* = 53	Individuals (Medical + Pharmacy Students) *N* = 164	Teams *N* = 61
Question					
Q2: Patient identifier		85 (77)	40 (75)	125 (76)	50 (82)
**Q8: Type of report**		**16 (14)**	**5 (9)**	**21 (13)** ^**†**^	1 (2)*
**Q9: Outcome attributed to adverse event**		**53 (48)**	**28 (53)**	**81 (49)**	**23 (38)**
Q11: Was this event associated with product use or treatment under Emergency Use Authorization (EUA)?		96 (86)	45 (85)	141 (86)	57 (93)
Q12: Describe the event, problem, or product use/medication error		88 (79)	44 (83)	132 (80)	54 (89)
**Q13_2: Relevant laboratory test(s)**		**11 (10)**	**7 (13)**	**18 (11)** ^**†**^	41 (67)*
Q13_3: Results of the above test(s)		11 (10)	3 (6)	14 (9)	1 (2)
Q13_7: Please provide the descriptive results of the test or procedure. Include results, analyses, and evaluations.		4 (4)	4 (8)	8 (5)	6 (10)
Q14: Other relevant history, including preexisting medical conditions (e.g., allergies, pregnancy, smoking and alcohol use, liver/kidney problems, etc.)		25 (23)	15 (28)	40 (24)	12 (20)
Q19_1: Name of suspect product		90 (81)	46 (87)	136 (83)	60 (98)
Q19_2: Strength of suspect product		4 (4)	3 (6)	7 (4)	46 (75)
Q19_3: Units for the strength of the suspect product		0 (0)	0 (0)	0 (0)	46 (75)
Q19_9: Frequency of administration of the suspect product		0 (0)	0 (0)	0 (0)	40 (66)
Q19_10: Route of administration of the suspect product		0 (0)	0 (0)	0 (0)	42 (69)
**Q21: Diagnosis for use (indication)**		**43 (39)**	**28 (53)**	**71 (43)** ^**†**^	42 (69)*
Q22: Product type (OTC, compounded, generic, biosimilar)		52 (47)	42 (79)	94 (57)	50 (82)
Q24: Event abated after use stopped or dose reduced?		47 (42)	23 (43)	70 (43)	38 (62)
Q25: Event reappeared after reintroduction?		70 (63)	29 (55)	99 (60)	47 (77)

Four questions (8, 9, 13_2, and 21) were tested for differences between the various groups using the chi-square test of independence

^†^ indicates a significant difference (*p* < 0.05) between individuals (medical + pharmacy students) and teams

* indicates a significant difference (*p* < 0.05) between medical students, pharmacy students, and/or teams

### Students’ perceptions of the activity

**Fig. 1:**
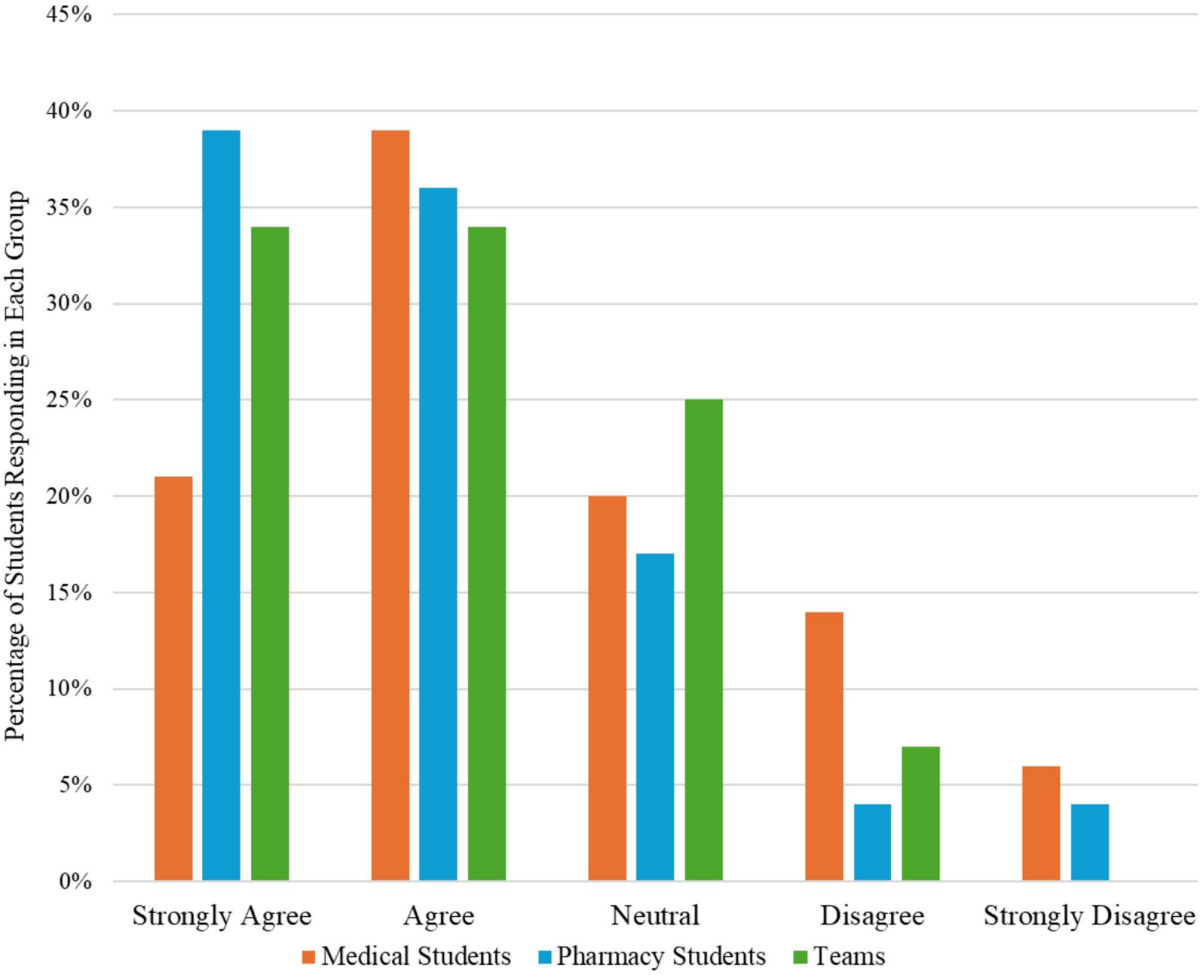
Responses to “practicing the reporting of an adverse drug event was a useful activity”. *When median responses to this question were compared between groups (using Dunn’s test), it was noted that Pharmacy Students felt the activity was more valuable than Medical Students (p-value = 0.015)

**Fig. 2:**
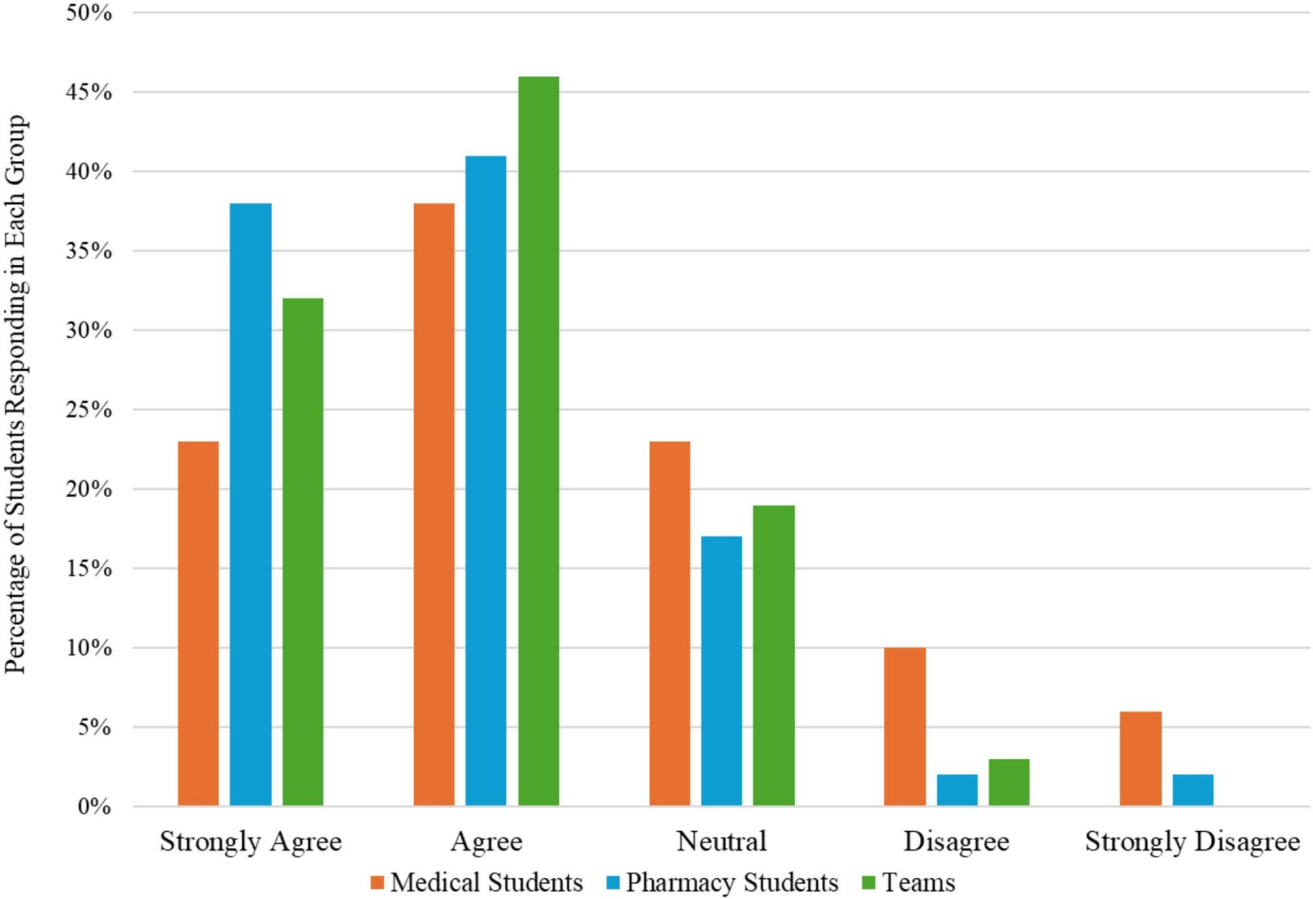
Responses to “after this activity, I feel more confident in reporting an adverse drug event”. *When median responses to this question were compared between groups (using Dunn’s test), it was noted that Pharmacy Students (p-value = 0.014) and Teams (p-value = 0.043) felt more confident reporting adverse drug reactions than Medical Students

Our assessment of median responses by group to the following statement: “Practicing the reporting of an adverse drug event was a useful activity” revealed significant differences among medical students, pharmacy students, and teams (*p* = 0.0095). Pharmacy students felt the activity was more valuable than medical students (*p* = 0.015, Fig. 1). Our assessment of median responses by group to the following statement: “After this activity, I feel more confident in reporting an adverse drug event” revealed that pharmacy students (*p* = 0.014) and teams (*p* = 0.043) felt more confident reporting an adverse drug event than medical students (Fig. 2).

## Discussion

This study demonstrates the impact of an interprofessional patient safety session in enhancing the completeness and accuracy of medication error reporting. Teams significantly outperformed individual medical and pharmacy students, underscoring the value of a collaborative approach in reporting medication errors. Interestingly, students and teams’ accuracy in selecting the type of report were low and teams did worse than individuals on this question. A more robust discussion of report types with future trainees will equip them with the knowledge needed for the selection of the most applicable type of report. This interprofessional learning experience not only showed greater team reporting performance compared with individuals but also boosted participants’ confidence in reporting, which is essential for fostering a culture of safety in clinical practice.

These findings are consistent with prior research emphasizing the benefits of IPE in enhancing students’ knowledge in patient safety competencies. Thom et al. highlighted that interprofessional activities promote better understanding of patient safety competencies and communication among medical, nursing, and pharmacy students [23]. Similarly, Gill et al. demonstrated that IPE fostered significant improvements in recognizing and addressing patient safety issues while focusing on communication errors, awareness of various disciplines’ roles and responsibilities, and strategies for addressing conflicts in teams [22]. Our study builds on this evidence by focusing specifically on medication error reporting—a less explored but critical competency in patient safety education. The 2023 systematic review by Grimes et al. underscored the importance of structured and rigorously evaluated interprofessional activities in medication safety education following the 3P model of planning, conducting (process), and evaluating (product) [25]. Our study aligns with the 3P model and complements their findings, showing that simulated team-based exercises can significantly enhance the accuracy and completeness of medication error reporting. By providing hands-on applications of the MedWatch form, this study adds practical value to the growing body of literature on the design and evaluation of IPE initiatives.

Educating and training healthcare professionals in reporting medication errors prior to practice is crucial. Our results highlight the impact of embedding medication error reporting training within health professional curricula and providing students with an interprofessional setting to practice error reporting. While Darbishire et al. discussed the value of having pharmacy students observe a practitioner reporting a medication error, our study proposes a more collaborative and active pedagogy [26]. By simulating the use of the MedWatch form, this study provides a replicable model, in Qualtrics, for other institutions to enhance student preparedness for real-world medication error reporting. The Erice Medication Errors Research Group’s (EMERGE, 2009) recommendations resonate with this study’s findings, particularly emphasizing the importance of (1) providing students with opportunities to practice skills that help reduce errors, (2) training all health-care professional students in mitigating medication errors [27].

This study suggests that collaborative reporting of medication errors, rather than reporting in silos, could ensure enhanced completion and accuracy of the report. Training teams to report collaboratively provides opportunities for mutual support, help with wording, collective responsibility, and increased accountability all of which could mitigate some of the barriers to reporting, such as time constraints, bystander effect, different reporting systems, and the desire for anonymity. Medical students’ lower confidence in error reporting compared with pharmacy students or teams warrants attention. This aligns with a study by Pirzadeh et al. that linked professional identity to medical students’ attitudes toward self-reporting. Enhancing medical students’ attitude and willingness to disclose medication errors through targeted interventions could be an essential medical education competency to address in advancing patient safety [28].

The study is subject to several limitations. For instance, medical students’ prior exposure to error reporting during their clinical rotations may have influenced their perceptions of this activity and performance compared to pharmacy students who lacked such experience at the time of the study. Additionally, the exclusion of a large portion of the initial sample due to incomplete data may limit the generalizability of the findings. Since the students were not awarded any points for completing the activity, they may have been less inclined to complete or submit the MedWatch form. Additionally, although we found no differences when comparing virtual versus in-person delivery methods, we acknowledge the lack of a true comparison group and the possibility of residual confounding affecting our findings. Future studies should aim to include larger, more balanced samples across health care disciplines to ensure broader applicability.

The findings of this study lay the groundwork for advancing the understanding and effectiveness of IPE focused on medication error reporting in health professions education. This preliminary data could set the stage for team-based reporting of medication errors in practice, particularly in settings with close interprofessional collaboration such as intensive care units or emergency departments. Future research could incorporate a mixed-method approach assessing the implementation of team-based error reporting and identifying factors that facilitate or hinder its applicability in practice such as team composition (i.e. one discipline vs. interprofessional, disciplines representation on the team…) and team dynamics.

## Conclusion

We demonstrated that a single interprofessional education session positively impacted completion rates and accuracy of medication error reporting when conducted by interprofessional teams as compared to individual health professional students. Educating health professional students on medication error reporting and providing an opportunity to practice reporting using a real-life case report was well accepted by students and has the potential to mitigate barriers by providing experience using a common reporting format. Future studies need to evaluate facilitators or barriers to implementing this approach.

## Electronic supplementary material

Below is the link to the electronic supplementary material.

Supplementary Material 1

## Data Availability

The datasets used and/or analyzed during the current study are available from the corresponding author on reasonable requests.

## Abbreviations

FDA: Food and Drug Administration
IPE: Interprofessional Education
MERP: Medication Error Reporting Program
ISMP: Institute for Safe Medication Practice
IRB: Institutional Review Board
N: Number
Q: Question
EMERGE: Erice Medication Errors Research Group
