# Supplementary material for: Enhancing medication error reporting through interprofessional education: analysis of Medwatch reporting accuracy and completion rates between teams and individuals
Source: BMC Med Educ. 2025 May 22;25:756. doi: 10.1186/s12909-025-07349-7 (PMC12100788; doi:10.1186/s12909-025-07349-7)
Supplement: Supplementary file 1 — Supplementary Material 1 [file 12909_2025_7349_MOESM1_ESM.docx]

Simulated MedWatch Voluntary Report

Start of Block: Default Question Block

Q46 I am a: (when individuals are completing the report)

- Medical Student (1)
- Pharmacy Student (2)

Q48 Please enter your team's number (when the team is completing the report)

________________________________________________________________

Q49 Please identify your scribe's discipline (when team is completing the report)

- Medicine (1)
- Pharmacy (2)

Q1 **About Patient**
* Required Information
For all other data fields please provide information, if available. Only fields with * are mandatory

Q2 **Patient Identifier**:
Please do NOT enter the Patient's Name or Social Security Number

________________________________________________________________

Q3 **Age or Date of Birth DOB:**
For the Age (please specify the unity of time for age in years, months, weeks, or days)
You can also enter the age as DOB in mm/dd/yyyy)

________________________________________________________________

Q4 **Gender**

- Female (1)
- Male (2)
- Intersex (3)
- Transgender (4)
- Prefer not to disclose (5)

Q5 **Weight and Unit** (lb or kg)

________________________________________________________________

Q6 **Ethnicity:** (check one)

- Asian (1)
- American Indian or Alaskan Native (2)
- Black or African American (3)
- White (4)
- Native Hawaiian or Other Pacific Islander (5)

End of Block: Default Question Block

Start of Block: Block 1

Q7 **About Problem**
* Required Information For all other data fields please provide information, if available. ONLY fields with * are mandatory.

Q8 **Adverse Event, Product Problem:**
Type of Report (Check all that apply).
 Note that definitions are provided after each type, for your reference.

- **Adverse Event:** It is not necessary to be certain of a cause/effect relationship between the adverse event and the use of the medical product(s) in question. Suspicion of an association is sufficient reason to report. Submission of a report does not constitute an admission that medical personnel or the product caused or contributed to the event. (1)
- **Product Use/Medication Error**: A product use/medication error includes confusion with labeling, packaging, product design; sound alike or look alike drug names; and device use that is inconsistent with the user's expectations or understanding. A product use/medication error is any preventable event that may cause or lead to inappropriate medication or device use or patient harm while the medication is in the control of the health care professional, patient, or consumer. (2)
- **Product Problem** (e.g., defects/malfunctions): Product quality problems include, for example: Suspected counterfeit product, suspected contamination, questionable stability, physical defects such as color, powdering, chipped; defective components for both devices or drug products, suspected super potent or subpotent medication. (3)
- **Problem with Different Manufacturer of Same Medicine**: Please include in the 'Describe Event, Problem, or Product Use Error' field, specific details relative to the switch between different manufacturers of the same medicine: the names of both manufacturers, length of treatment on each product, product strength, and a clinical description of the expected clinical response and how it was changed. If you have returned to your prior medication, please note if the prior therapeutic response has returned. (4)

Q9 **Outcome Attributed to Adverse Event**:
(Check all that apply)

- Death (Date of Death) (mm/dd/yyyy) (1) __________________________________________________
- Life-threatening (2)
- Hospitalization (initial or prolonged) (3)
- Disability or Permanent Damage: Check if the adverse event resulted in a substantial disruption of a person's ability to conduct normal life functions. Such would be the case if the adverse event resulted in a significant, persistent or permanent change, impairment, damage or disruption in the patient's body function/structure, physical activities and/or quality of life. (4)
- Congenital Anomaly/Birth Defects (5)
- Other Serious or Important Medical Events (6)
- Required Intervention to Prevent Permanent Impairment/Damage (7)

Q10 **Date of Event** (mm/dd/yyyy):

________________________________________________________________

Q11 **Was this event associated with a product use or treatment under Emergency Use Authorization (EUA)?**
EUA: Under section 564 of the Federal Food, Drug, and Cosmetic Act, the FDA Commissioner may allow unapproved medical products or unapproved uses of approved medical products to be used in an emergency to diagnose, treat, or prevent serious or life-threatening diseases or conditions caused by chemical, biological, radiological and nuclear threat agents when there are no adequate, approved, and available alternatives. For more information, click here

- Yes (1)
- No (2)

Q12 **Describe Event, Problem, or Product Use/Medication Error:**
 Click to see more information about Describe Event, Problem or Product Use/Medication Error
  
(Reports should include "[Product name] use for COVID-19 under Emergency Use Authorization (EUA):" at the beginning of the "Describe Event" field. This text will be repopulated when you select the "Yes" radio button". Please modify [Product name] to reflect the specific product you are reporting on.)

 Total of **4000** characters allowed.

________________________________________________________________

Q13 **Relevant Tests/Laboratory Data**:

- **Test Date** (mm/dd/yyyy): (1) __________________________________________________
- **Test Name:** Laboratory test, imaging study, or tissue analysis performed to help diagnose disease or monitor treatment. For example Chest X-ray, Serum Creatinine, Lung Biopsy, Blood glucose, etc. (2) __________________________________________________
- **Test Result**: The value of the Laboratory Test Result. Report more descriptive results under ‘Additional Comments’ (3) __________________________________________________
- **Test Unit**: Units of measure of the Laboratory Test Result. (4) __________________________________________________
- **Low Test Range:** Low end of the normal range for the Laboratory Test. (5) __________________________________________________
- **High Test Range**: High end of normal range for the Laboratory Test. (6) __________________________________________________
- **Additional Comments**: Please provide the descriptive results of the Test or Procedure. Include the results, analyses, and evaluations. Total of 2000 characters allowed. (7) __________________________________________________

Q14 **Other Relevant History, Including Preexisting Medical Conditions**:
(e.g., allergies, pregnancy, smoking and alcohol use, liver/kidney problems, etc.)

Total of 2000 characters allowed.

________________________________________________________________

Q15 **Product Available for Evaluation?**
(Do not send the product to FDA)

- Yes (1)
- No (2)
- Returned to Manufacturer on (mm/dd/yyyy) (3) __________________________________________________

Q16 **Do you have a picture of the product?**
 *(check yes if you are including a picture)*

- Yes (1)

End of Block: Block 1

Start of Block: Block 2

Q18
**About Product**
* Required Information For all other data fields please provide information, if available. ONLY fields with * are mandatory
 This section is to report about products involving cosmetic, dietary supplement or food/medical food. To report about a medical device only, you can skip to questions 26.

Q19 **Suspect Product:**

- **Name***: Use the trade/brand name, generic name, or the name of each ingredient in a combination product. For example, "Augmentin" or "amoxicillin and clavulanate potassium" (1) __________________________________________________
- **Strength:** Strength is the ingredient amount in each tablet or capsule, the concentration of an injectable or solution, etc. There may be more than one ingredient amount in a combination product. For example, "800mg/160mg" or "20mg" (2) __________________________________________________
- **Unit**: Please select the unit(s) for the strength. For example, mg (milligram), ml (milliliter), mg/mg, mg/ml, etc. (3) __________________________________________________
- **Manufacturer/Compounder**: When reporting a product quality problem or therapeutic failure for a generic drug product, it is helpful in evaluating the report that the name of the manufacturer, the strength of the drug, the NDC number and the lot number be provided. This field is not a mandatory field, but it can be useful for evaluation if available. (4) __________________________________________________
- **NDC # or Unique ID:** The national drug code (NDC #) is found on the original product label and/or packaging, but is usually not found on dispensed pharmacy prescriptions. NDC numbers are useful to the FDA in investigating drug product quality problems. Please include any zeros and dashes in the NDC # as they appear on the label and/or packaging. This field is not a mandatory field, but it can be useful for evaluation if available. (5) __________________________________________________
- **Lot #:** When reporting a product quality problem or therapeutic failure for a generic drug product, it is helpful in evaluating the report that the lot number be provided. This number is usually not found on dispensed pharmacy prescriptions, but on the original product label and/or packaging. Consider speaking with the pharmacist/pharmacy that dispensed the product to obtain that information. This field is not a mandatory field, but it can be useful for evaluation if available. (6) __________________________________________________
- **Dose or Amount:** Please enter the amount of the product that is taken each time. For example, if 1 tablet, enter 1, if 2 puffs, enter 2, if 5 drops, enter 5, if 5mg, enter 5, etc. (7) __________________________________________________
- **Unit**: Please enter a unit of measure after entering a Dose or Amount. For products with more than one ingredient, use Dosage Form-DF. (1 teaspoon = 5 mL; 1 Tablespoon = 15 mL; 1 Ounce = 30 mL) (8) __________________________________________________
- **Frequency**: Daily, BID, TID, QID, HS, PRN, Other (9) __________________________________________________
- **Route** (10) __________________________________________________

Q20 **Treatment Dates/Therapy Dates:**
(give best estimate of length of treatment (start/stop) or duration.)

- **Therapy started on**: (mm/dd/yyyy) (1) __________________________________________________
- **Therapy stopped on**: (mm/dd/yyyy) (2) __________________________________________________
- **Duration** with units (years, months, weeks, days) (3) __________________________________________________
- **Is therapy still on-going**? Answer with yes or no (4) __________________________________________________

Q21 **Diagnosis for Use** (indication) :

________________________________________________________________

Q22
**Product Type**: (Check all that apply)

- **OTC:** Over-the-counter (OTC) drug products are those drugs that are available to consumers without a prescription. OTC drugs generally have these characteristics: their benefits outweigh their risks, the potential for misuse and abuse is low, consumer can use them for self-diagnosed conditions, they can be adequately labeled, and health practitioners are not needed for the safe and effective use of the product. (1)
- **Compounded:** This checkbox should not be checked (1) if the medication is an FDA approved product from a manufacturer that is dispensed at a pharmacy or (2) when the medication is an FDA approved product that is admixed or reconstituted according to the product’s labeling (i.e. admixed IV antibiotics). FDA approved products can be found in the Orange Book. Compounding is a practice in which a licensed pharmacist, licensed physician and/or outsourcing facility combines, mixes, or alters ingredients of a drug to create a medication tailored to the medical needs of an individual patient. (2)
- **Generic**: A generic drug is a medication manufactured to be the same as an existing approved brand-name drug in dosage form, safety, strength, route of administration, quality, and performance characteristics. (3)
- **Biosimilar:** Biosimilars are biological products that are demonstrated to be interchangeable or have no clinically significant differences from an FDA-approved biological reference product. (4)

Q23 **Expiration Date** (mm/dd/yyyy)
(If you only know the month and year, default to the last day of the month.)

________________________________________________________________

Q24 **Event Abated After Use Stopped or Dose Reduced?**
If available, this information is particularly useful in the evaluation of a suspected adverse event. In addition to checking the appropriate box, please provide supporting lab tests and dates, if available, in the narrative field for relevant test/laboratory data on the About Problem screen.

- Yes (1)
- No (2)
- Doesn't apply (3)

Q25 **Event Reappeared After Reintroduction?**
If available, this information is particularly useful in the evaluation of a suspected adverse event. In addition to checking the appropriate box, please provide supporting lab tests and dates, if available, in the narrative field for relevant test/laboratory data on the About Problem screen.

- Yes (1)
- No (2)
- Doesn't apply (3)

End of Block: Block 2

Start of Block: Block 3

Q26 **About Device**
* Required Information For all other data fields please provide information, if available. ONLY fields with * are mandatory.
If you do not need to report about devices, you may skip this page. Insert how to skip this page. 

Q27 **Suspect Medical Device:**

- **Brand Name**: This information might be on a label attached to a durable device, or be on a package of a disposable device, or appear in labeling materials of an implantable device. (1) __________________________________________________
- **Common Device Name**: The generic or common name of the suspect medical device or a generally descriptive name (e.g., urological catheter, heart pacemaker, patient restraint). Please do not use broad generic terms such as "catheter", "valve", "screw", etc. (2) __________________________________________________
- **Procode:** The FDA classification product code (a code that consists of three alpha characters) that corresponds to the common device name can be entered here if it is known. (3) __________________________________________________

Q28 **Manufacturer:**

- Name (1) __________________________________________________
- Country (2) __________________________________________________
- City (3) __________________________________________________
- State/Province/Region (4) __________________________________________________
- Zip/Postal code (5) __________________________________________________
- Model #: The exact model number found on the device label or accompanying packaging. (6) __________________________________________________
- Catalog #: The exact number as it appears in the manufacturer's catalog, device labeling, or accompanying packaging. (7) __________________________________________________
- Serial #: This number can be found on the device, its label, or accompanying packaging; it is assigned by the manufacturer, and should be specific to each device. (8) __________________________________________________
- Lot #: This number can be found on the label or packaging material. (9) __________________________________________________
- Unique Identifier (UDI) #: This number can be found on the device, its label, or accompanying packaging. The number is located below the barcode and begins with one of the following three elements: (01), +, =. Record all numbers, letters, parentheses, and symbols included in the UDI Number. For more information regarding the UDI Number, refer to the UDI webpage or contact the UDID HelpDesk at UDIDSupport@fda.hhs.gov. (10) __________________________________________________
- Expiration Date (mm/dd/yyyy): If available, this date can often be found on the device itself or printed on the accompanying packaging. (If you only know the month and year, default to the last day of the month.) (11) __________________________________________________

Q29 **Operator of Device:**

- Health Professional (1)
- Patient/Consumer (2)
- Other (3)

Q30 **If Implanted, Give Date** (mm/dd/yyyy):

________________________________________________________________

Q31 **If Explanted, Give Date** (mm/dd/yyyy):

________________________________________________________________

Q32 **Is this a single-use device that was reprocessed and reused on a patient?**

- Yes (1)
- No (2)

Q33 **If Yes to item above, Enter Name and Address of Reprocessor**

- Name (1) __________________________________________________
- Country (2) __________________________________________________
- Address Line 1: (3) __________________________________________________
- Address Line 2: (4) __________________________________________________
- City (5) __________________________________________________
- State/Province/Region (6) __________________________________________________
- Zip/Postal code: (7) __________________________________________________

Q34 **Was this device serviced by a third party servicer?**
 Third Party Servicers and Independent Service Organizations (ISOs) ("Third Party Servicers", "ISOs", or "Third Party Entities"): These are entities, other than the manufacturer or healthcare establishments, that maintain, restore, refurbish, or repair a finished device after distribution, for purposes of returning it to the safety and performance specifications established by the manufacturer and to meet its original intended use.

- Yes (1)
- No (2)
- Unknown (3)

End of Block: Block 3

Start of Block: Block 4

Q35 **About Concomitant Products Help for Concomitants**
* Required Information For all other data fields please provide information, if available. ONLY fields with * are mandatory.


Information on the use of concomitant products can frequently provide insight into previously unknown interactions between products, or provide an alternative explanation for the observed adverse event

Q36 **Product names and therapy dates** (Exclude treatment of event):

- Product 1 Name (1) __________________________________________________
- Therapy Start Date (mm/dd/yyyy) (2) __________________________________________________
- Therapy End Date (mm/dd/yyyy) (3) __________________________________________________
- Product 2 Name (4) __________________________________________________
- Therapy Start Date (mm/dd/yyyy) (5) __________________________________________________
- Therapy End Date (mm/dd/yyyy) (6) __________________________________________________

End of Block: Block 4

Start of Block: Block 5

Q37 **Reporter:**
 FDA recognizes that confidentiality is an important concern in the context of adverse event reporting. The patient's identity is held in strict confidence by FDA and protected to the fullest extent of the law. However, to allow for timely follow-up in serious cases, the reporter's identity may be shared with the manufacturer unless specifically requested otherwise. FDA will not disclose the reporter's identity in response to a request from the public, pursuant to the Freedom of Information Act.

Q38 **Name and Address**

- Name (First and Last Name) (1) __________________________________________________
- Address (2) __________________________________________________
- City (3) __________________________________________________
- State (4) __________________________________________________
- Zip Code (5) __________________________________________________
- Phone # (6) __________________________________________________
- Email address (7) __________________________________________________

Q39 **Health Professional?**

- Yes (1)
- No (2)

Q40 **Occupation:**
 Please indicate your occupation (particularly type of health professional), and include specialty, if appropriate.

________________________________________________________________

Q41 **Also reported to:**
 
Please indicate whether you have also notified or submitted a copy of this report to the manufacturer and/or distributor of the product, or, in the case of medical device reports only, to the user facility (institution) in which the event occurred. This information helps to track duplicate reports in the FDA database.

- Manufacturer (1)
- User Facility (2)
- Distributer/Importer (3)

Q42 **If you do NOT want your identity disclosed to the manufacturer, please check the below box**

- I do NOT want my identity disclosed to the manufacturer (1)

Q43 **Submission of a report does not constitute an admission that medical personnel or the product caused or contributed to the event.**

End of Block: Block 5

Start of Block: Block 6

Q44 Practicing the reporting of an adverse drug event was a useful activity

- Strongly agree (1)
- Agree (2)
- Neither agree nor disagree (3)
- Disagree (4)
- Strongly disagree (5)

Q45 After this activity, I feel more confident in reporting an adverse drug event

- Strongly agree (1)
- Agree (2)
- Neither agree nor disagree (3)
- Disagree (4)
- Strongly disagree (5)

Q47 Whose responsibility is it to report an adverse drug event?

- Prescribing healthcare provider (1)
- Dispensing Pharmacist (2)
- Individual who observed the error (4)
- Individual who is aware of the error (5)
- Patient (3)
- All of the above (6)

End of Block: Block 6
